# Fish bone and chitosan based nanogels in green dentistry: sustainable and eco-friendly biomimetic remineralizing agents for early enamel caries

**DOI:** 10.1186/s12903-026-08008-z

**Published:** 2026-03-27

**Authors:** Hend El-Messiry, Nada Atef, Eman Alaa, Dalia I. Sherief, Iman A. Fathy

**Affiliations:** 1 Oral Biology, Faculty of Dentistry, Ain Shams University, Cairo, Egypt; 2 Pedodontics and Dental Public Health, Faculty of Dentistry, Ain Shams University, Cairo, Egypt; 3 Pedodontics and Dental Public Health, Faculty of Oral and Dental Medicine, Future University in Egypt, Cairo, Egypt; 4 Biomaterials, Faculty of Dentistry, Ain Shams University, Cairo, Egypt; 5 Biomaterials, School of Dentistry, Badya University, Giza, Egypt

**Keywords:** Biomimetic remineralization, Early enamel caries, Nano fish-bone gel, Nano-chitosan gel, Green dentistry, Sustainable dental materials, SEM–EDX analysis

## Abstract

**Background:**

Biomimetic enamel reconstruction represents an innovative strategy in restorative dentistry and materials science, providing a biologically and eco-friendly inspired approach for managing early enamel caries. This study aimed to evaluate the remineralizing potential of organically derived biomimetic nanogels—chitosan nanogel (n-C) and fish bone nanogel (n-FB)—on demineralized enamel, using scanning electron microscopy (SEM), and energy dispersive X-ray (EDX) analysis for semi-quantitative calcium/phosphate assessment.

**Methods:**

Fifty-seven extracted primary molars were demineralized for 96 h. Three samples were used as the demineralized control group, while the remaining 54 were randomly assigned to three groups: [1] Artificial Saliva (AS) group—specimens incubated in artificial saliva at 37 °C for seven days; [2] Nano-Fish Bone (n-FB) group—n-FB gel applied once daily for five minutes, rinsed, then incubated in refreshed artificial saliva; [3] Nano-Chitosan (n-C) group—n-C gel applied similarly. Specimens were analysed using SEM and EDX for surface morphology and elemental Ca/P ratio, and data were statistically analysed.

**Results:**

Both biomimetic nanogels significantly reversed the demineralization effects, showing enamel prism reformation. For the calcium-to-phosphorus (Ca/P) ratio, the highest mean value was recorded in the AS group (2.77 ± 0.06), followed by the NC group (2.58 ± 0.61), the n-C group (2.45 ± 0.18), and the lowest in the n-FB group (2.19 ± 0.38). ANOVA analysis demonstrated a significant difference between groups (*P* = 0.020). The n-C group and n-FB produced a more uniform and well-mineralized surface structure closest to normal enamel.

**Conclusions:**

Both nanogels provided enhanced remineralization, however, nano–fish bone exhibited more uniform surface morphology and significantly higher phosphate levels than nano–chitosan, suggesting a comparatively greater remineralization potential.

## Background

Dental caries remain one of the most prevalent chronic diseases globally and continue to represent a major public health concern. It is a dynamic process characterized by alternating cycles of demineralization and remineralization at the tooth surface [1]. When the balance shifts toward demineralization, subsurface enamel dissolution occurs, leading to the formation of white spot lesions (WSLs), the earliest visible sign of enamel caries. Early enamel caries is highly prevalent in pediatric patients, particularly in deciduous teeth, which are more susceptible to rapid lesion progression due to their thinner and less mineralized enamel [2]. These lesions are clinically significant as they are reversible at this initial stage, and successful remineralization can restore enamel integrity and prevent cavitation [3].

Conventional preventive measures rely mainly on fluoride therapy and calcium–phosphate-based biocomposites such as casein phosphopeptide–amorphous calcium phosphate (CPP–ACP) and bioactive glass. Although these materials exhibit remineralizing potential, they have limitations related to their penetration depth, ion release control, and their inability to fully replicate the hierarchical microstructure of natural enamel [4].

Biomimetic remineralization has emerged as a promising minimally invasive strategy for the management of early carious lesions. This approach seeks to replicate the natural biomineralization process by guiding the nucleation and oriented growth of hydroxyapatite crystals that resemble those of native enamel [5]. Advances in nanotechnology and tissue engineering have facilitated the development of biomimetic materials capable of rebuilding the hierarchical microstructure of enamel. Among these, nanohydroxyapatite (nHA) has demonstrated remarkable efficacy owing to its close structural and chemical resemblance to natural enamel apatite. These biomimetic systems promote the formation of well-ordered hydroxyapatite crystals and regulate crystal growth through biomineralization mediators, thereby restoring enamel integrity and function in a manner that closely mimics natural processes [6, 7]. 

Natural materials such as egg shells, fish bones and chitosan provide a sustainable route for producing hydroxyapatite (HA) used in enamel remineralization [8–11]. Harvesting HA from these renewable sources reduces reliance on synthetic materials and minimizes chemical waste, energy use, and environmental impact. This approach aligns with the principles of green dentistry, offering safe, effective, and eco-conscious materials for preventive and restorative dental care [12]. 

Chitosan, a naturally occurring polysaccharide derived from crustacean shells, has gained attention as a potential agent for enamel remineralization due to its biocompatibility and biodegradability. Its positively charged molecules allow it to adhere to the negatively charged enamel surface, enhancing the deposition of calcium and phosphate ions and promoting the formation of new mineral layers. When used in combination with other remineralizing agents such as fluoride or nanohydroxyapatite, chitosan has shown a synergistic effect, improving both mineral content and mechanical properties of demineralized enamel [13–15]. Beyond mineral deposition, its antibacterial and film-forming properties further support enamel protection, making chitosan a promising component in minimally invasive strategies for early caries management [16]. 

Fish bones represent an abundant and sustainable source of calcium phosphate minerals, particularly calcium, phosphate, and carbonate, which are essential constituents of hydroxyapatite (HA) [11]. Due to their natural composition, fish bones can be processed to produce biocompatible HA that closely resembles the mineral phase of human enamel and dentin. Hydroxyapatite derived from fish bones has demonstrated favourable physicochemical properties, including high crystallinity, porosity, and bioactivity, making it suitable for applications in bone tissue engineering, dental restorations, and remineralization strategies [17, 18]. 

Although chitosan exhibits remineralizing potential, its nanoparticulate form shows bioadhesiveness and a tendency to agglomerate, which may limit increasing its concentration beyond 1% without affecting dispersion. Moreover, chitosan is primarily amorphous or semi-crystalline and does not inherently provide a mineral phase [19]. In contrast, fish bone–derived nanoparticles consist of crystalline hydroxyapatite similar to enamel mineral and can be incorporated at higher concentrations (3%) without compromising structural stability [20]. This higher mineral content and crystallinity may enhance ion release and support improved remineralization potential.

Thus, the current study aimed to investigate the remineralizing potential of two organic biomimetic waste nanomaterials [fish bone (n-FB) and Chitosan (n-C)] on demineralized enamel using SEM and EDX for quantitative calcium/ phosphate assessment.

The hypothesis tested was that no significant difference in enamel remineralization (as measured by Ca/P ratio using EDX and surface morphology using SEM) was detected before and after application of fish bone–derived nanohydroxyapatite or chitosan nanomaterials gels on demineralized enamel samples.

## Materials and methods

### Sample size

The sample size calculation was performed using G*Power software (version 3.1.9.7), guided by data obtained from a previous investigation [21] employing an ANOVA: fixed effects, omnibus, one-way design. The analysis was conducted to ensure adequate statistical power for detecting overall differences among the experimental groups. The parameters were set at an alpha level of 0.05 and a beta level of 0.05, providing a power of 95%. An effect size corresponding to a Cohen’s d of 0.55 derived from prior findings, was used in the computation. The power analysis indicated that a minimum total sample size of 57 specimens would be sufficient to identify statistically significant differences in the percentage of mineral deposition among the groups.

### Preparation of enamel specimens

Fifty-seven archived extracted human teeth (sound second primary molars) were obtained from the tooth bank of the Pedodontics department, faculty of dentistry, Ain Shams university within a period of 4 months. All teeth had been extracted previously for clinical reasons unrelated to the present study and were anonymized prior to use. As this study used archived anonymized teeth, informed consent was waived. Ethical approval for the study was granted by the Research Ethics Committee, Faculty of Dentistry, Ain Shams University (FDASU-REC ER 102422), in accordance with the principles of the Declaration of Helsinki. After extraction, the teeth were cleaned of soft-tissue remnants and stored in 0.4% thymol solution at 4 °C and used within one month. Teeth with caries, cracks, restorations, or developmental defects were excluded.

A standardized 3 × 3 mm² window was outlined on the mid-buccal enamel surface of each tooth using a permanent marker. All remaining crown surfaces were then coated with two layers of acid-resistant nail varnish (Florelle Nail Polish; Milan, Italy), leaving only the marked window exposed. The varnish was allowed to dry completely before further procedures. Each tooth was subsequently incubated at 37 °C for 48 h in individual test tubes containing freshly prepared artificial saliva (pH 6.57). The artificial saliva formulation consisted of sterile deionized water (99.6%), potassium chloride (0.12%), sodium chloride (0.08%), magnesium chloride (0.01%), carboxymethyl cellulose (0.10%), dibasic potassium phosphate (0.03%), and calcium chloride (0.01%).

### Remineralizing agents preparation and characterization

The nano chitosan gel (n-C) was prepared at Nano Gate, Cairo, Egypt. To prepare the nanoparticles, 1 g of chitosan powder was dissolved in 200 ml of 1% acetic acid (pH = 4) and stirred for six hours to ensure complete homogenization. Afterward, 150 ml of a 0.2% w/v Tripolyphosphate (TPP) solution was added gradually, causing the previously clear mixture to become turbid, indicating nanoparticles formation. The resulting suspension was purified by centrifugation (Hermle Z32 HK, Germany) at 12,000 rpm for 30 min, repeated three times using deionized water. For 1% w/v gel formulation, 100 mg of the chitosan nano powder was dispersed in 10 ml of distilled water and subjected to sonication and continuous stirring for one hour to obtain a 10 mg/ml dispersion. Subsequently, 1 g of hydroxypropyl methylcellulose (Loba Chemie, India) was slowly incorporated into the mixture under gentle heating and vigorous agitation until a uniform, smooth paste was achieved.

The nano chitosan gel was characterized using transmission electron microscopy (TEM) to evaluate particle size and morphology. TEM analysis was performed with a JEOL JEM-2100 high-resolution instrument operating at 200 kV. A drop of the nanoparticle suspension was placed onto a Formvar-coated 300-mesh copper grid (Ted Pella, Inc.) and left to dry under ambient conditions before imaging. Particle dimensions and size distribution were determined using specialized image-analysis software. Chemical functional groups were identified using Fourier-transform infrared spectroscopy (FTIR) with a Vertex 70 RAM II spectrometer (Bruker), allowing confirmation of the characteristic bonds associated with chitosan nanoparticles.

As for the nano fish bone gel (n-FB), it was prepared in accordance with a previous study [22] using Nile Tilapia bones obtained from a local market. The bones were cleaned by boiling for two hours to remove residual organic matter and then left to air-dry at room temperature. After drying, they were calcined in an electric furnace at 900 °C for five hours and allowed to cool for 15 min. The resulting ash was ground into a fine powder and further milled using a planetary ball mill (PM-400) at 350 RPM for 10 h with intermittent 3-minute cycles to obtain nanoparticles. To formulate a 3% w/v gel, 0.3 g of this nano powder was dispersed in 10 ml of deionized water, after which 1 g of hydroxypropyl methylcellulose (Loba CHIME, India) was gradually added under gentle heating and vigorous stirring until a uniform gel was formed. The prepared nanoparticles were then characterized using several analytical techniques.

Particle size and morphology were examined by TEM (Talos F200i, Thermo Scientific) operating at 200 kV, with samples prepared by placing a drop of the colloidal suspension on a Formvar-coated 300-mesh copper grid and allowing it to dry. Functional groups were identified using FT-IR spectroscopy (Vertex 70 RAM II, Bruker), while crystallinity was assessed through X-ray diffraction using an XPERT-PRO diffractometer within a 2θ range of 20°–80°, a minimum step size of 0.001, and Cu Kα radiation (λ = 1.54614 Å).

### Demineralization and remineralization procedures

Each tooth was stored individually in a sealed container containing approximately 10 mL of demineralizing solution and kept at 37 °C for 96 h. The solution consisted of 50 mM acetic acid adjusted to pH 4.5, 2.2 mM calcium nitrate, 2.2 mM monobasic potassium phosphate, 5.0 mM sodium azide, and 0.5 ppm sodium fluoride, with pH adjustment performed using NaOH. A fresh demineralizing solution was provided every 24 h. Following the four-day demineralization period, the teeth were rinsed thoroughly and subjected to ultrasonic cleaning in distilled deionized water three times (5 min each) to ensure complete termination of the process [23]. 

Three teeth were reserved as the negative control (NC) group for demineralized enamel surface imaging and elemental analysis using EDX. The remaining 54 samples were randomly divided into three groups (*n* = 18 each):

Artificial Saliva (AS) group: Teeth were stored in artificial saliva at 37 °C throughout the experimental period without receiving any treatment. Each tooth was stored individually in a sealed container containing approximately 10 mL of artificial saliva. The saliva medium was replaced daily to maintain consistent ionic conditions and prevent saturation.

Nano Chitosan Gel (n-C) group: The n-C gel was applied once daily for 5 min. After application, the gel was removed, the teeth were rinsed with distilled water and then stored individually in 10 mL artificial saliva at 37 °C for seven days. The saliva was renewed every 24 h [22]. 

Nano Fish Bone Gel (n-FB) group: The n-FB gel was applied using the same regimen as the n-C group—daily application for 5 min followed by rinsing and incubation in artificial saliva at 37 °C for one week, with daily replacement of the saliva [24]. 

### Scanning electron microscopy and EDX analysis

Teeth were examined using a ThermoFisher Quattro S Field Emission Gun scanning electron microscope (Apex™ Standard Version 1.3, AMETEK Inc., USA). Imaging was performed in low-vacuum mode (20–200 Pa) using a secondary electron detector (LVD) at an accelerating voltage of 15 kV, spot size equal to 3 and a working distance of 16.3 mm, with a chamber pressure of 65 Pa. Each tooth was rinsed with distilled water to remove any residues, excess fluid was blotted with lint-free tissue and stored in a desiccator prior to imaging. Specimens were mounted on aluminium stubs using carbon adhesive tabs. SEM imaging was performed to assess surface morphology, texture, and the density of the sample surface. Elemental composition, specifically calcium (Ca) and phosphorus (P) content, was determined using EDX analysis. The Ca/P ratio was calculated to evaluate the extent of remineralization.

**Fig. 1:**
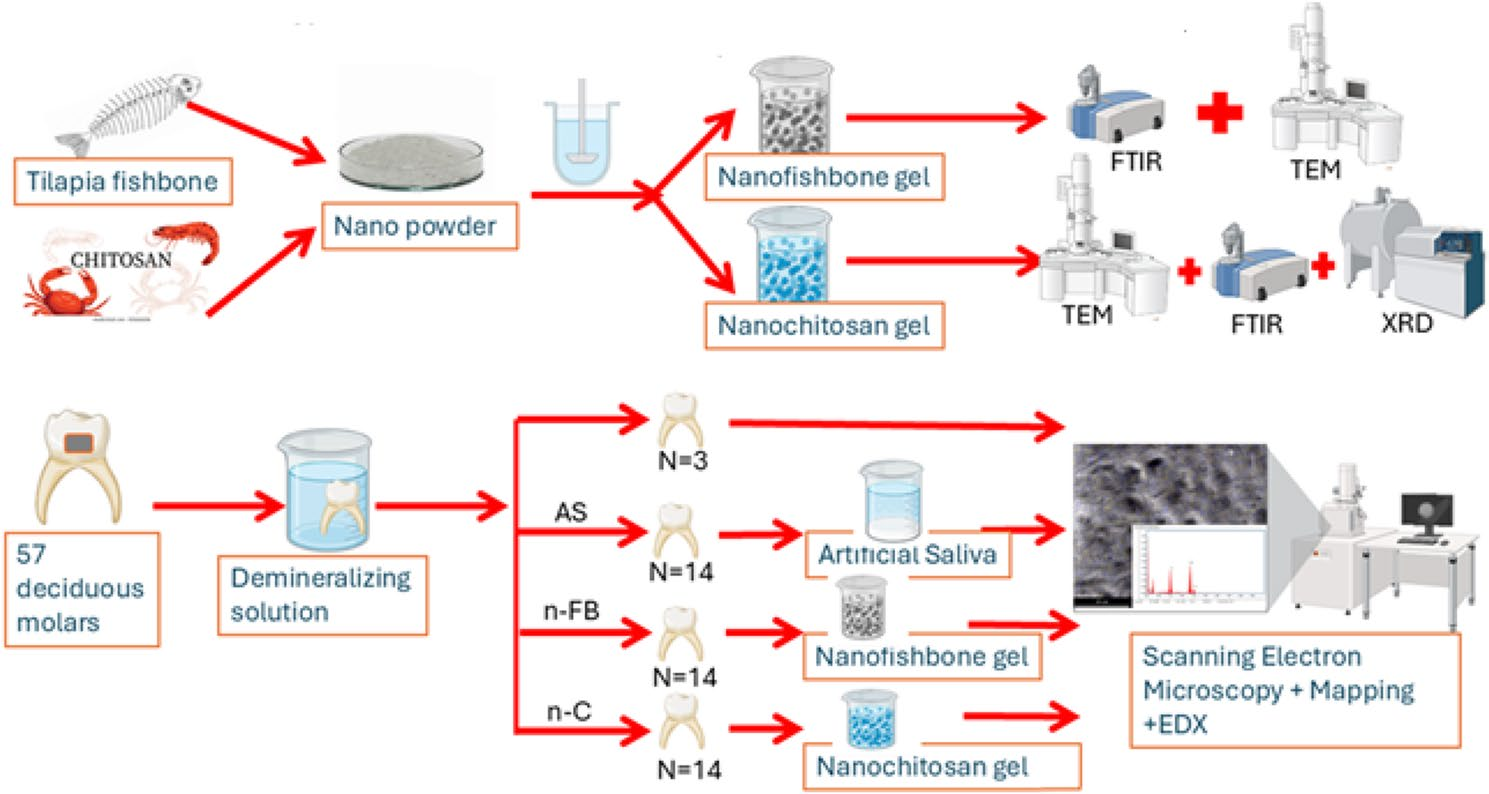
Schematic illustration of the experimental design

A schematic illustration of the overall experimental design is presented in Fig. 1.

### Statistical analysis

Elemental data for calcium and phosphorus obtained from SEM-EDX mapping were organized in spreadsheets and summarized as mean values with their corresponding ranges. The distribution of the data was evaluated using the Kolmogorov–Smirnov test, which confirmed that the variables followed a normal distribution. Accordingly, one-way ANOVA was applied to assess differences among the study groups, followed by Tukey’s post hoc test for multiple pairwise comparisons.

A significance threshold of *p* ≤ 0.05 was adopted for all analyses. Statistical processing was performed using SPSS software version 23.0 (SPSS Inc., Chicago, IL, USA) for Windows.

## Results

### Characterizations of nanogels

#### TEM imaging

**Fig. 2:**
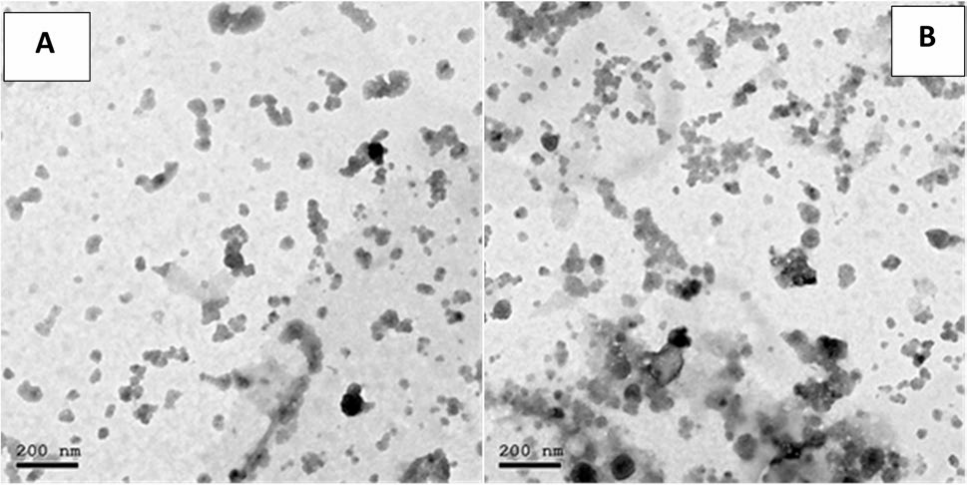
**A** & **B**: TEM images of the prepared n-Chitosan

TEM imaging of chitosan nanoparticles demonstrated that they were mainly spherical with slight irregularities and showed uniform dispersion. Their average diameter was approximately 45 ± 5 nm, consistent with nanoscale calcium phosphate materials. Mild particle agglomeration was evident, which is typical for high–surface-energy nanostructures. The measured d-spacing values matched the characteristic lattice planes of hydroxyapatite, confirming the formation of well-ordered phosphate-based nanomaterials (Fig. 2A, B).

**Fig. 3:**
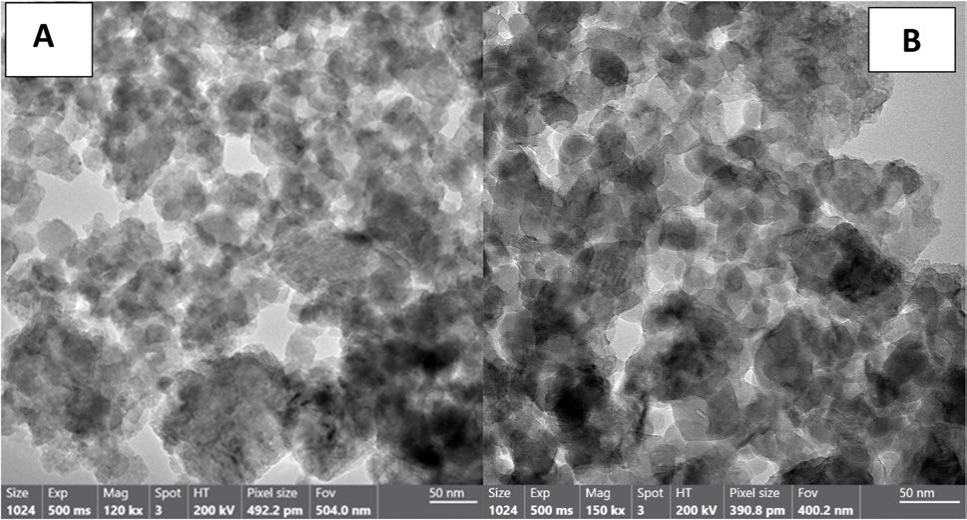
**A**, **B**: TEM images of the prepared n-FB particles

TEM analysis of the fish bone-derived nanoparticles revealed clusters of agglomerated particles with predominantly irregular to quasi-spherical shapes. Individual particle diameters ranged from approximately 10 to 30 nm, consistent with the high surface area and reactivity expected from biomineral-based nanomaterials. Moderate agglomeration was observed, likely driven by van der Waals forces and surface energy effects. High-magnification images (120 kx) displayed internal lattice contrast within several particles, indicative of crystallinity, which correlates with the sharp reflections detected in the XRD patterns. These structural characteristics suggest efficient nanoscale conversion of fish bone apatite, potentially enhancing its bioactivity and dissolution behaviour for biomedical or environmental applications (Fig. 3A, B).

#### FTIR results

**Fig. 4:**
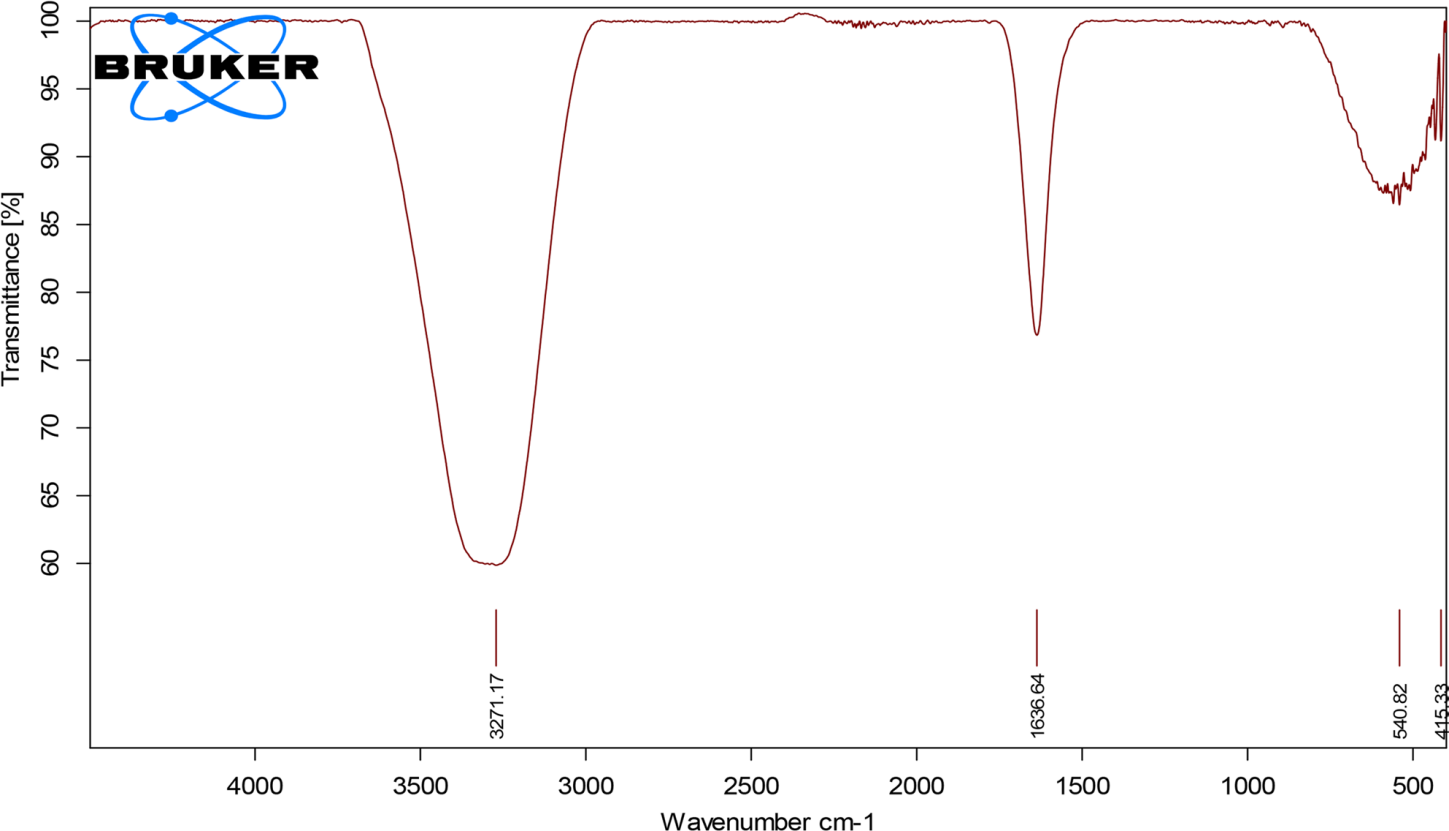
FTIR spectrum of chitosan nanoparticles

The FTIR spectrum of nano chitosan particles showed the characteristic phosphate vibrational bands, verifying calcium phosphate formation. Peaks at 1035 and 1092 cm⁻¹ corresponded to PO₄³⁻ asymmetric stretching (ν₃), while the band at 962 cm⁻¹ indicated symmetric stretching (ν₁). Bending modes (ν₄ and ν₂) at 602, 565, and 470 cm⁻¹ further supported phosphate group presence. Carbonate-related signals (1415–1444 cm⁻¹) suggested slight CO₃²⁻ incorporation possibly due to atmospheric exposure or partial substitution in the phosphate matrix. The broad O–H region near 3425 cm⁻¹ and weak C–H peaks around 2925 cm⁻¹ indicated surface-bound water or minimal organic residues. Overall, the spectrum confirmed a crystalline phosphate-based material with minor carbonate substitution (Fig. 4).

**Fig. 5:**
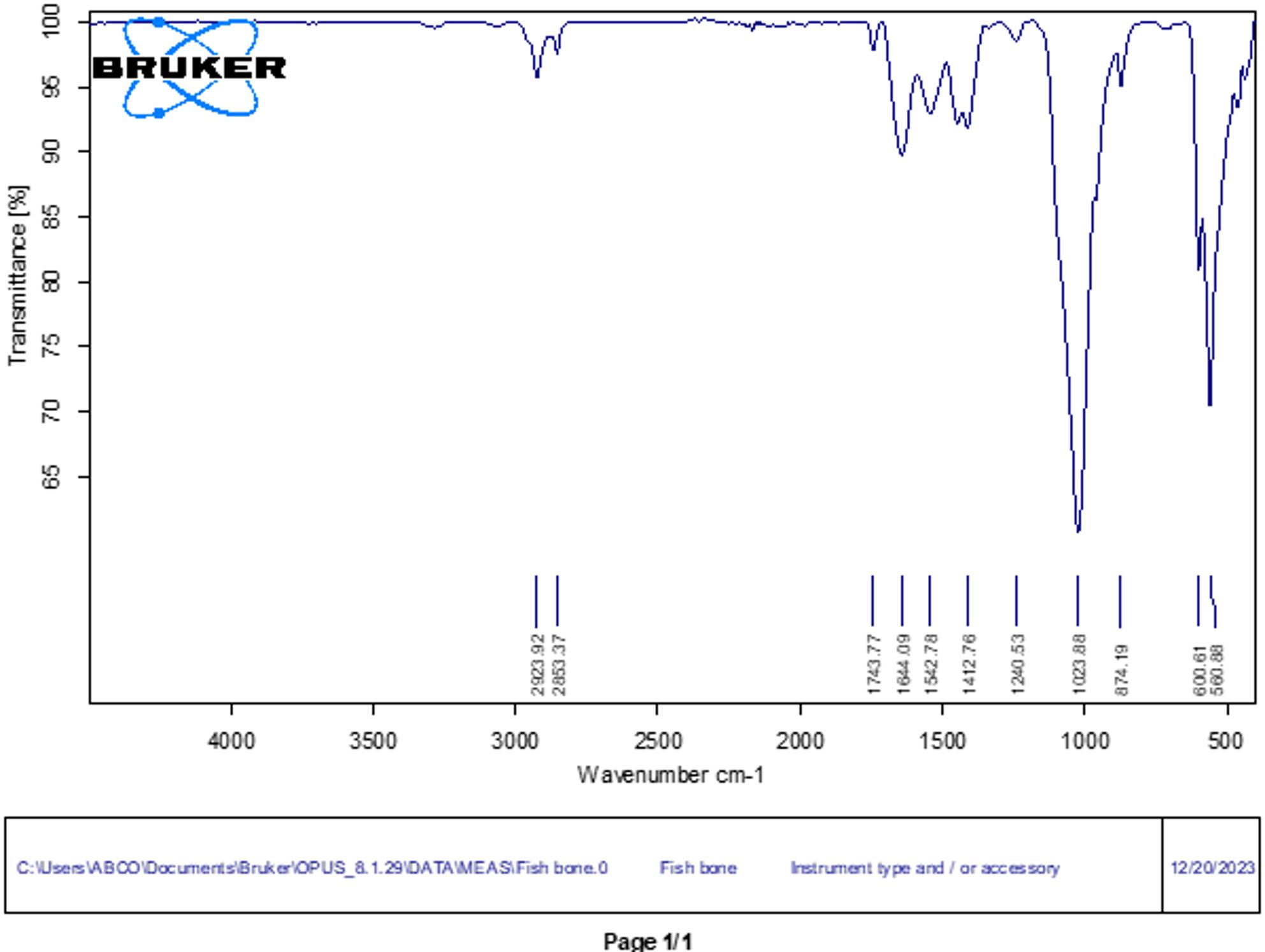
FTIR spectrum of fishbone nanoparticles

The FTIR spectrum of the fish bone nanoparticles confirmed the presence of functional groups characteristic of calcium phosphate, particularly hydroxyapatite. Strong absorption peaks at 1035 and 1092 cm⁻¹ were attributed to asymmetric stretching (ν₃) of PO₄³⁻, while the band at 962 cm⁻¹ corresponded to symmetric stretching (ν₁). Bending vibrations (ν₄) appeared at 602 and 565 cm⁻¹, with the skeletal deformation mode (ν₂) at 470 cm⁻¹, all indicative of phosphate groups typical of hydroxyapatite. Minor bands at 1415–1444 cm⁻¹ suggested the presence of carbonate (CO₃²⁻), reflecting partial B-type substitution common in biological apatite. Broad absorption around 3425 cm⁻¹ and weaker peaks near 2925 cm⁻¹ corresponded to O–H and C–H stretching, likely due to adsorbed water and residual organics. These spectral features confirm the formation of a bone-derived calcium phosphate material with structural properties resembling natural hydroxyapatite (Fig. 5).

#### XRD analysis

**Fig. 6:**
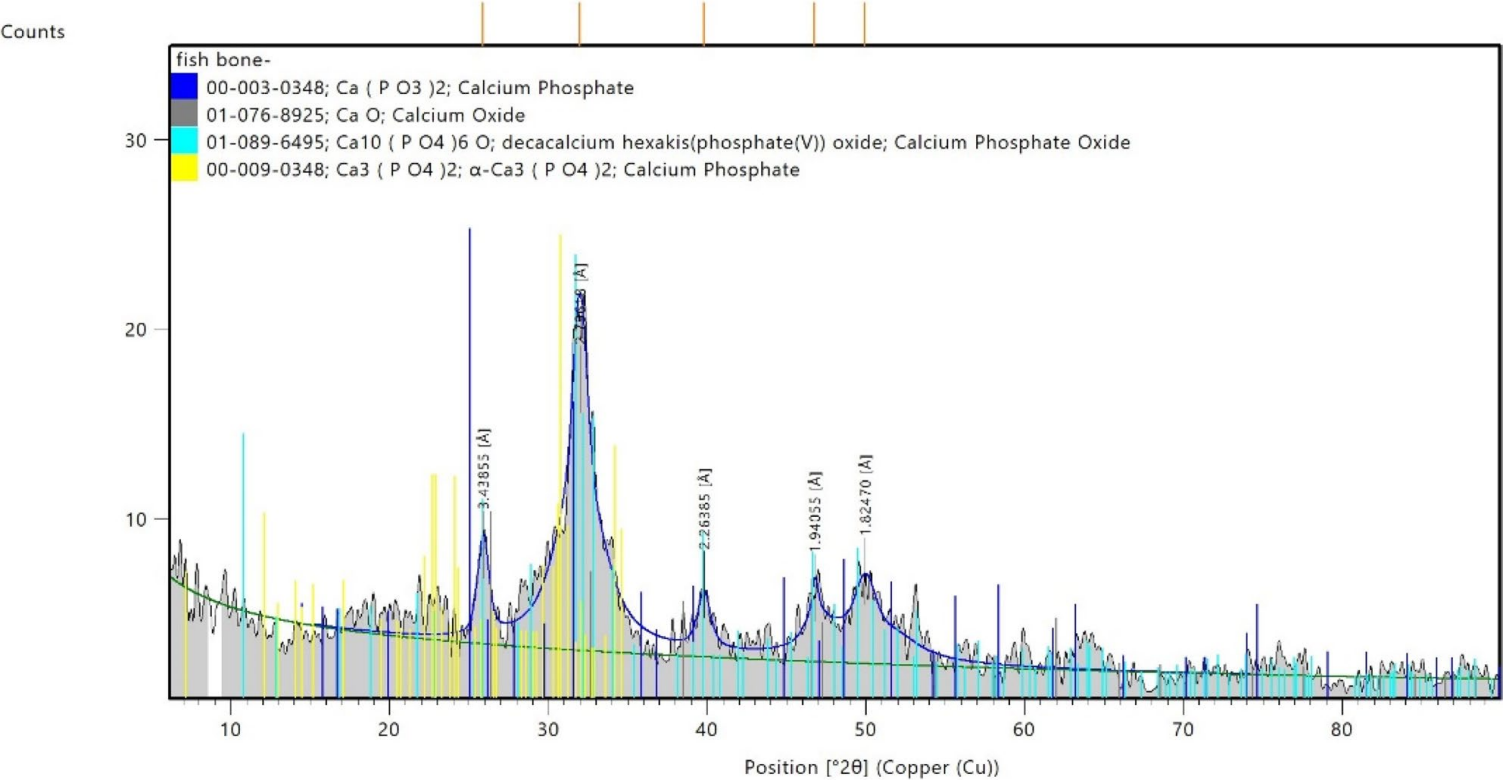
XRD of fish bone nanoparticles

The X-ray diffraction (XRD) pattern of the synthesized fish bone nanoparticles (Fig. 6) demonstrated a highly crystalline structure with reflections characteristic of multiple calcium phosphate phases. Prominent peaks between 25° and 35° 2θ, particularly at ~ 31.8°, correspond to hydroxyapatite [Ca₁₀(PO₄)₆(OH)₂] as per JCPDS card no. 00-003-0438. Additional phases detected included tricalcium phosphate (Ca₃(PO₄)₂, JCPDS no. 00-009-0348), calcium oxide (CaO, JCPDS no. 01-078-9625), and Decacalcium hexakis (phosphate(VI)) oxide (Ca₁₀(PO₄)₆O, JCPDS no. 01-089-6495), indicating a multiphasic composition. The presence of CaO likely reflects partial thermal decomposition of organic components and phosphate matrices during calcination. The sharp and intense diffraction peaks confirm the material’s high crystallinity, while minor peak broadening suggests nanoscale particle dimensions. The coexistence of hydroxyapatite with other calcium phosphate phases is typical of bio-derived calcined materials and highlights the heterogeneous nature of the fish bone matrix.

#### SEM evaluation of enamel surface morphology

**Fig. 7:**
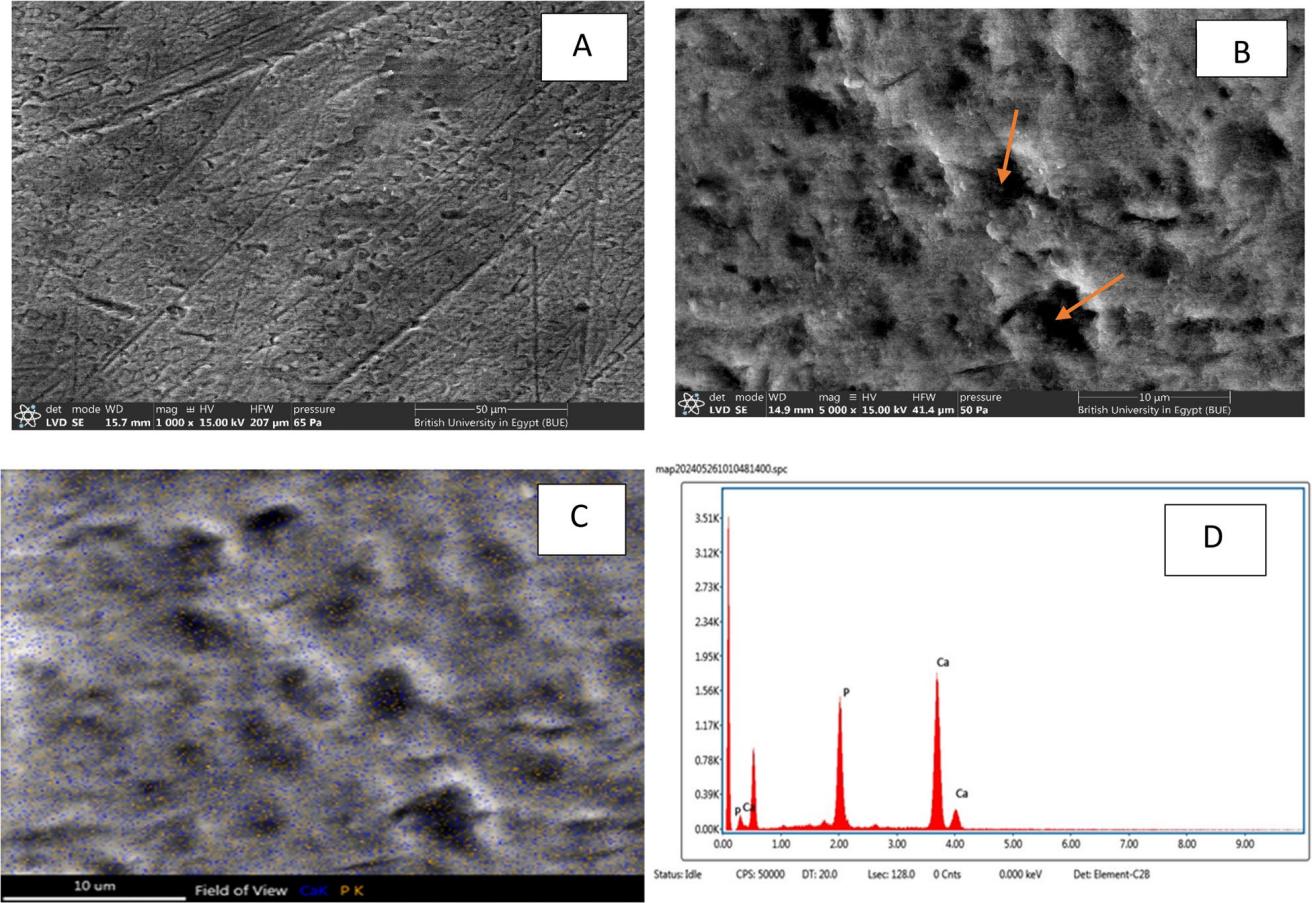
**A**, **B**: SEM of negative control group (demineralized enamel) at magnification 1000x & 5000x showing demineralized enamel with rough surface irregularities, porosities and lost crystals (red arrows). **C**, **D**: SEM-EDX elemental mapping for Ca and P

Negative Control group: SEM micrographs of the demineralized enamel at 1000x &5000x reveal a markedly irregular and porous surface(red arrows), characterized by prominent variable patterns of enamel prisms dissolution involving body, tail, and inter-prismatic substance with loss of minerals & crystals in core of the prisms & also revealed surface irregularities confirming the effectiveness of the demineralization protocol (Fig. 7A, B).

**Fig. 8:**
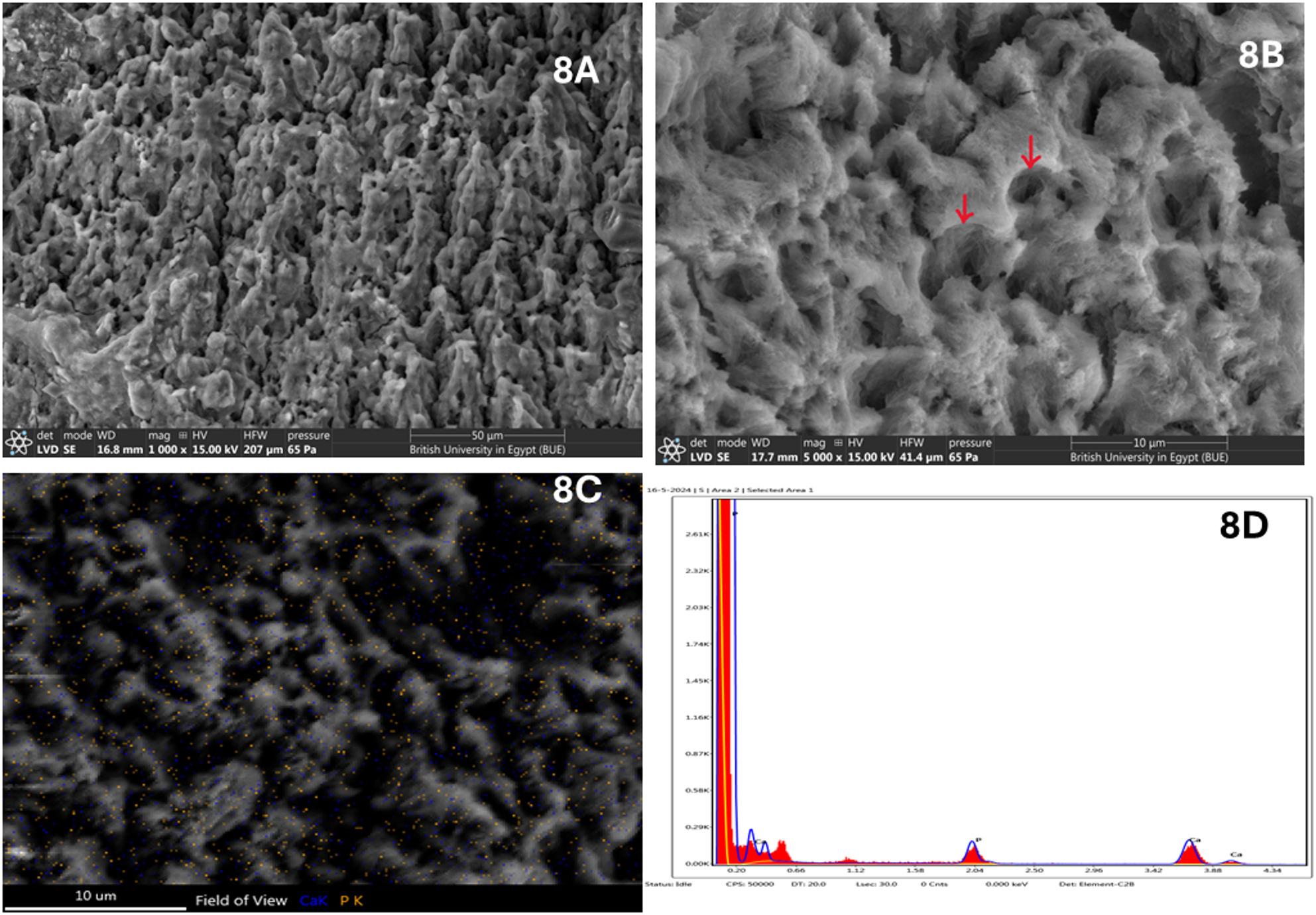
**A**, **B**: SEM of the artificial saliva (AS) group at magnifications 1000x & 5000x showing partial remineralization of the peripheries of some prisms and the core of others (red arrows), (**C**, **D)**: SEM-EDX elemental mapping for Ca and P

Artificial saliva (AS) group: SEM micrographs of enamel specimens stored in artificial saliva at 1000x &5000x magnifications show partial surface recovery compared with the demineralized control. The enamel surface appears less irregular, with some reduction in surface roughness and partial filling of interprismatic voids. However, multiple porosities and shallow cavities remain visible, indicating that the surface repair is incomplete and uneven. The enamel structure lacks the compact and continuous morphology characteristic of fully remineralized enamel (Fig. 8A, B).

**Fig. 9:**
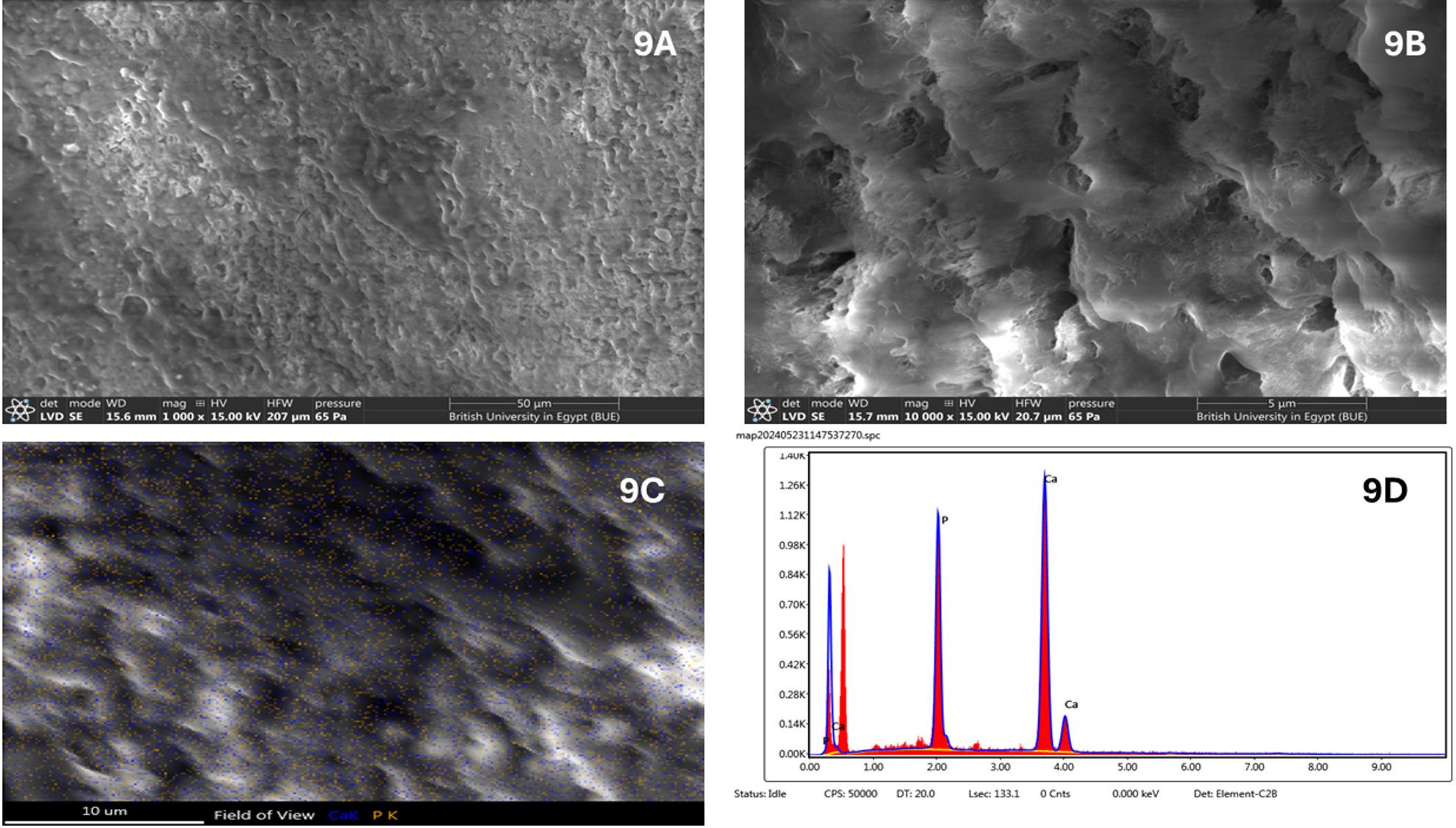
**A**, **B**: SEM of nano Chitosan gel (n-C) group at magnifications 1000x & 5000x showing a new dense layer of apatite. **C**, **D**: SEM-EDX elemental mapping for Ca and P

Nano Chitosan gel (n-C) Group: SEM micrographs of enamel treated with nano-chitosan gel at 1000x &5000x magnifications show a noticeable improvement in surface morphology compared with both the demineralized and artificial saliva groups. The enamel surface appears smoother and more consolidated, with partial masking of interprismatic defects and reduced surface porosity. A relatively continuous layer covering the enamel surface can be observed, suggesting deposition of remineralizing material facilitated by the chitosan matrix. Despite this improvement, minor surface irregularities and shallow depressions are still present, indicating incomplete structural restoration (Fig. 9A, B).

**Fig. 10:**
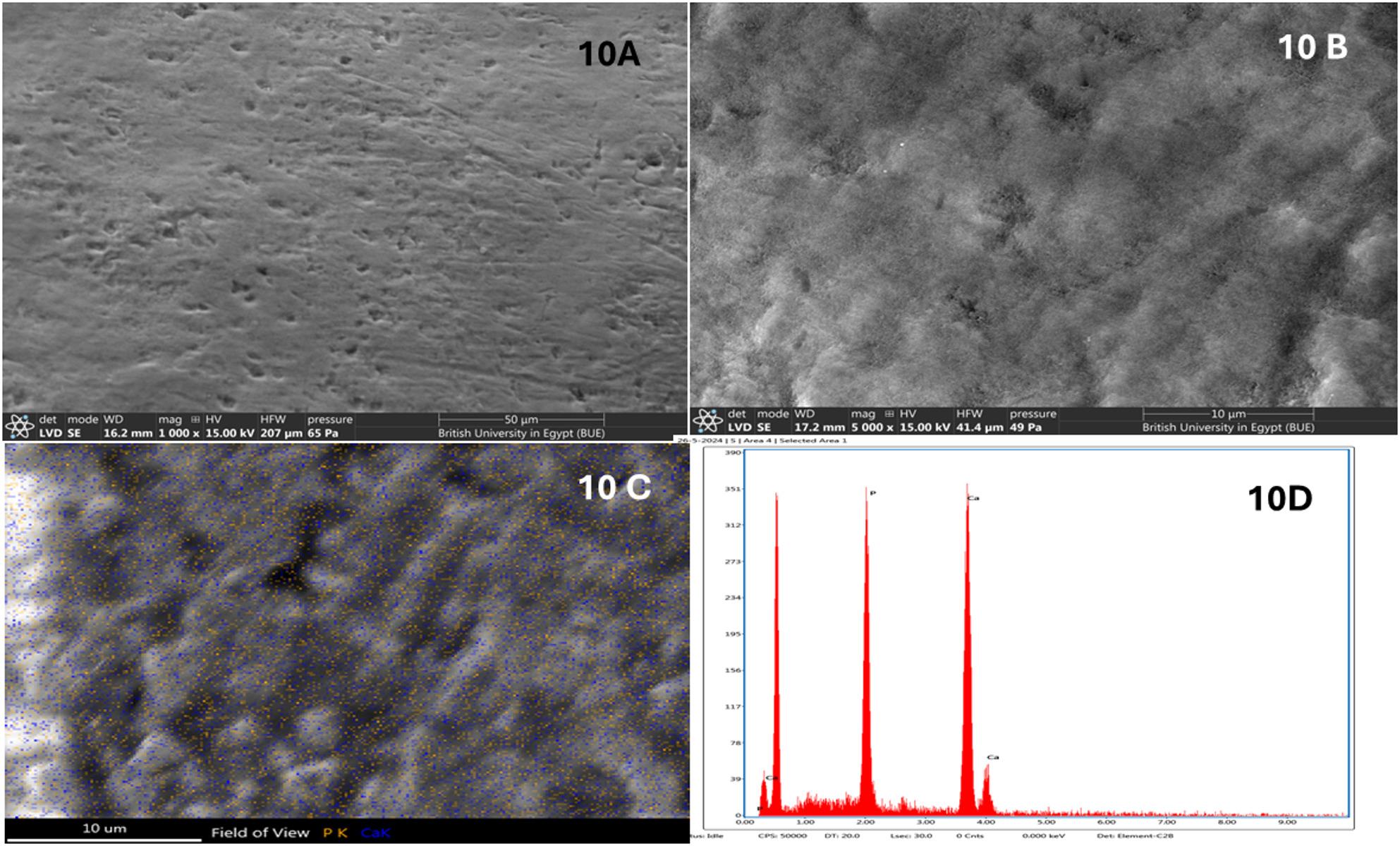
**A**, **B**: SEM of nano fish bone gel (n-FB) Group at magnifications 1000x & 5000x showing homogenous enamel surface. **C**, **D**: SEM-EDX elemental mapping for Ca and P

Nano Fish Bone gel (n-FB) Group: SEM micrographs at 1000x &5000x magnifications reveal a markedly dense and uniform surface morphology. The enamel surface appears highly compact with substantial reduction in surface porosity and near-complete masking of interprismatic defects with remineralization of hydroxyapatite crystals in core and peripheries of prisms (Fig. 10A, B).

#### Energy dispersive X-ray (EDX) quantitative analysis of enamel specimens

EDX analysis revealed that the highest mean phosphorus (P) content was observed in the Fish Bone (n-FB) group (31.83 ± 4.81), followed by the Chitosan (n-C) group (29.05 ± 1.51), the Negative Control (NC) group (26.57 ± 1.16), and the Artificial Saliva (AS) group (26.55 ± 0.43). One-way ANOVA indicated a statistically significant difference among the groups (*P* = 0.001). Post hoc comparison using Tukey’s test showed no significant difference between the NC and AS groups (Table 1; Fig. 1). Regarding the mean calcium (Ca) content no significant difference was detected among the different groups (Table 1).

**Table 1:** Mean ± SD values, results of ANOVA & post hoc tests for the comparison between different groups regarding Ca, P and Ca/P ratio

	NC Group	(AS)Group	*n*-C Group	*n*-FB Group	F-test	*p*-value
Ca						
Mean ± SD	68.36 ± 15.47	73.45 ± 0.43	70.95 ± 1.51	68.17 ± 4.81	0.843	0.480
P						
Mean ± SD	26.57 ± 1.16^C^	26.55 ± 0.43^C^	29.05 ± 1.51^B^	31.83 ± 4.81^A^	8.425	0.001*
Ca/P ratio						
Mean ± SD	2.58 ± 0.61^A^	2.77 ± 0.06^A^	2.45 ± 0.18^B^	2.19 ± 0.38^B^	3.756	0.020*

Significance level *p* ≤ 0.05

*significant

Tukey’s post hoc: Means sharing the same superscript letter are not significantly different

For the calcium-to-phosphorus (Ca/P) ratio, the highest mean value was recorded in the AS group (2.77 ± 0.06), followed by the NC group (2.58 ± 0.61), the n-C group (2.45 ± 0.18), and the lowest in the n-FB group (2.19 ± 0.38). ANOVA analysis demonstrated a significant difference between groups (*P* = 0.020). Tukey’s post hoc test indicated no significant difference between NC and AS groups, nor between the n-C and n-FB groups (Table 1).

## Discussion

Fish bone waste generated by the food industry can be valorized into bioactive calcium phosphates, supporting sustainable resource utilization and circular economy strategies [25]. Naturally derived materials such as fish bone hydroxyapatite and chitosan provide biomimetic alternatives to conventional remineralizing agents, including fluoride, CPP-ACP, and synthetic nano-hydroxyapatite [26, 27]. The incorporation of marine biowaste–derived biomaterials into dental applications is therefore consistent with the principles of green dentistry and sustainable material development [28, 29].

The demineralization protocol was conducted over 96 h, with daily renewal of the solution to maintain a stable pH of 4.2 and avoid saturation of the demineralizing medium [30]. 

SEM–EDX analysis is a reliable approach for evaluating enamel remineralization, as it combines detailed surface morphology assessment with quantitative and spatial elemental analysis of calcium and phosphorus, enabling comprehensive evaluation of mineral recovery and distribution [31, 32]. 

Including both negative (demineralized enamel) and positive control groups (AS group) is essential for validating in vitro enamel remineralization studies. A negative control of demineralized enamel provides a baseline outcome against which remineralization can be compared, confirming the extent of mineral loss induced by the demineralization protocol itself and enabling accurate assessment of recovery. While a positive control, such as specimens stored in artificial saliva without active treatment, models the natural remineralizing environment of the oral cavity and allows assessment of the extent to which simple salivary buffering contributes to spontaneous mineral gain. Positive controls ensure that any additional effects seen in treatment groups exceed the background benefit of saliva-mediated remineralization [33] .The concentration of the nano–fish bone gel (3%) was selected according to a prior study that demonstrated effective enamel remineralization at this concentration. In contrast, the nano-chitosan gel was prepared at 1% based on a pilot study, as chitosan exhibits high viscosity and effective ion-binding capacity at lower concentrations. Increasing chitosan concentration beyond this level adversely affects gel handling and surface penetration.

TEM analysis confirmed successful nanoscale formation of both materials with distinct structural characteristics. Nano-chitosan particles appeared predominantly spherical with relatively uniform dispersion and mild agglomeration, consistent with polymer-based nanostructures. Their nanoscale size may enhance surface interaction and ion-binding capacity, supporting remineralization processes [34]. In contrast, fish bone–derived nanoparticles exhibited smaller particle sizes (10–30 nm), irregular morphology, and visible lattice fringes, indicating crystalline apatite structure. The presence of nanoscale crystalline hydroxyapatite suggests increased surface reactivity and bioactivity, which may contribute to improved mineral deposition [35]. 

FTIR spectra of both nano-chitosan and fish bone–derived nanoparticles showed characteristic phosphate vibrational bands corresponding to hydroxyapatite, confirming the formation of calcium phosphate phases. The presence of carbonate bands indicates partial lattice substitution typical of biological apatite. Broad O–H absorption further supports features consistent with hydrated apatite structures. Overall, the spectral profiles suggest that both nanomaterials retain chemical characteristics similar to natural hydroxyapatite, which may contribute to their bioactivity and remineralization potential [35]. 

XRD analysis confirmed that the nano–fish bone material was predominantly composed of crystalline hydroxyapatite, as indicated by characteristic diffraction peaks within the 25–35° 2θ range. Minor secondary calcium phosphate phases were also detected, reflecting the multiphasic nature typical of calcined biogenic apatite. The sharp reflections demonstrate high crystallinity, while slight peak broadening indicates nanoscale crystallite dimensions. Such structural characteristics are associated with increased surface reactivity, which may enhance remineralization potential [20]. 

The SEM–EDX mapping findings demonstrate a clear progression in enamel remineralization among the studied groups. Artificial saliva provides a source of calcium and phosphate ions and simulates the buffering capacity of natural saliva, allowing some degree of mineral redeposition on the demineralized enamel surface [36]. However, this process relies primarily on diffusion-driven ion availability without active carriers or nucleation promoters, resulting in slow, superficial, and often non-uniform mineral deposition. Consequently, while artificial saliva can reduce surface porosity and initiate early mineral gain, it is generally insufficient to achieve substantial structural repair or deep lesion remineralization when used alone.

In contrast, treatment with nano-chitosan gel (n-C group) produced a more compact and organized enamel surface, indicating enhanced mineral deposition. This was found in agreement with many studies which stated that enhanced remineralization effect is largely attributable to chitosan’s unique bioadhesive and chelating properties. Chitosan possesses multiple cationic amine groups that facilitate strong adhesion to the negatively charged enamel surface, promoting prolonged contact between the biomaterial and the lesion site [37]. This bioadhesion increases local retention of calcium and phosphate ions, creating a supersaturated microenvironment that encourages mineral nucleation and growth within demineralized enamel prisms [38]. The combined effect of prolonged surface retention and ion chelation accelerates the re-deposition of hydroxyapatite-like mineral phases, allowing for more effective surface repair compared with passive remineralization. Chitosan was also found to inhibit the demineralization process and increase pH in acidic medium [10]. 

The nano-fish bone gel demonstrated the most pronounced remineralization, showing a dense, homogeneous enamel surface approaching those of sound enamel. The superior performance of the fish bone nanogel may be explained by its biomimetic calcium-phosphate composition, high bioavailability of hydroxyapatite, crystallinity and ability to closely replicate the natural mineral phase of enamel, thereby enabling deeper lesion infiltration and stable mineral integration [22]. 

As for the quantitative assessments for calcium and phosphate levels, the higher phosphate concentrations observed in the nano-fish bone and nano-chitosan groups, can be explained by the central role of phosphate ions in the early stages of enamel remineralization. Phosphate availability is a key determinant for hydroxyapatite nucleation, as it governs the initial formation of amorphous calcium phosphate and its subsequent transformation into crystalline phases [39]. 

Biomimetic systems such as fish-bone–derived hydroxyapatite and chitosan-based matrices thus provide phosphate-rich environments and promote phosphate stabilization at the enamel surface, thereby enhancing mineral precipitation. Artificial saliva, while containing phosphate ions, relies mainly on passive diffusion, resulting in lower and less consistent phosphate uptake [40]. 

In contrast, calcium ions are typically abundant in experimental media and quickly reach equilibrium at the specimen surface, resulting in relatively stable calcium levels across groups [41]. The absence of a statistically significant difference in calcium concentration among demineralized, chitosan-treated, and fish-bone–treated groups also appear a confusing finding; however, it can be attributed to the rapid equilibration and surface re-adsorption of calcium ions. Following demineralization, exposed negatively charged sites on the dentine/enamel surface readily attract calcium ions from the surrounding media, thus surface calcium can still be detected by EDX even in the absence of intact hydroxyapatite crystals [42]. 

The variation in Ca/P ratios among the studied groups reflects differences in the nature and quality of mineral deposition, rather than the absolute presence of calcium alone. The highest Ca/P ratio observed in the artificial saliva suggests preferential surface accumulation of calcium ions with relatively limited phosphate incorporation [40]. This phenomenon is consistent with saliva-mediated remineralization, which relies mainly on passive diffusion and often results in calcium-rich but structurally immature mineral phases.

Similarly, the lack of a significant difference between the AS and negative control (NC) groups indicates that surface calcium adsorption can occur even in the absence of effective remineralization, leading to elevated Ca/P ratios without true restoration of enamel mineral architecture.

In contrast, the lower Ca/P ratios recorded in the chitosan (n-C) and fish-bone (n-FB) groups indicate enhanced phosphate incorporation and formation of calcium-phosphate phases closer to the stoichiometry of biological hydroxyapatite. During early remineralization, biomimetic systems are known to favour the formation of phosphate-rich precursor phases (amorphous calcium phosphate) that progressively transform into more stable hydroxyapatite [43]. The absence of a significant difference between the n-C and n-FB groups suggests that both materials promote a similar remineralization pathway characterized by improved mineral quality rather than superficial calcium deposition. The significantly lower Ca/P ratio in the FB group further supports the formation of phosphate-rich, biologically relevant mineral phases, which are considered indicative of more effective and structurally integrated remineralization. Overall, these findings demonstrate that a lower Ca/P ratio reflects superior remineralization efficacy compared to higher ratios dominated by surface calcium accumulation.

Based on the above findings from SEM and EDX the tested null hypothesis was rejected.

Although this study was limited to surface evaluation using SEM and EDX, this approach was intentionally selected to address the primary aim of the investigation, which was to confirm the remineralizing potential of nano fish-bone and chitosan gels. Surface morphology and elemental composition provide direct and sensitive indicators of early mineral deposition, allowing rapid verification of whether these biomimetic agents can initiate remineralization. Establishing this proof of concept is a critical first step before undertaking more complex analyses to quantify mineral depth, crystallinity, or mechanical recovery. In addition, the in vitro design does not account for the complex oral environment, including salivary dynamics, biofilm activity, and mechanical stresses, which may influence remineralization behaviour in vivo.

Future studies are recommended to complement surface elemental analysis to better characterize the remineralization induced by these agents. Techniques such as X-ray diffraction (XRD) and Fourier-transform infrared spectroscopy (FTIR) should be employed to evaluate crystal structure and phase composition. Additionally, microhardness testing and nanoindentation could assess functional recovery of the remineralized tissue. Direct comparative studies between fish bone– and chitosan-based nanogels and well-established remineralizing agents such as CPP-ACP, nano-hydroxyapatite, and fluoride-containing materials are also recommended. Such comparative investigations would allow objective benchmarking of the biomimetic and sustainable nanogels against conventional therapies.

## Conclusion

Within the limitations of this study, both fish bone– and chitosan-based nanogels significantly enhanced enamel remineralization compared with the control groups, as demonstrated by SEM surface morphology and EDX elemental analysis. The nano–fish bone gel exhibited more uniform surface mineralization. These findings confirm the remineralizing capability of both biomimetic nanogels on early enamel lesions.

## Data Availability

The datasets during and/or analysed during the current study available from the corresponding author on reasonable request.

## Abbreviations

WSL: White spot lesions
HA: Hydroxyapatite
nHA: Nanohydroxyapatite
NC: Negative control
n-C: Nano-chitosan gel
n-FB: Nano-fishbone gel
AS: Artificial saliva
